# New Considerations in the Design of Clinical Trials for Bone Metastases

**DOI:** 10.4021/wjon445w

**Published:** 2012-02-19

**Authors:** Madeline Lemke, Karen Lien, Liang Zeng, Marko Popovic, Michelle Zhou, Julia Digiovanni, Emily Chen, Edward Chow

**Affiliations:** a Rapid Response Radiotherapy Program, Department of Radiation Oncology, Odette Cancer Centre, Sunnybrook Health Sciences Centre, University of Toronto, Canada

**Keywords:** Bone metastases, Palliative radiotherapy, Consensus, Future direction

## Abstract

Palliative radiotherapy (RT) is prescribed to patients with bone metastases to alleviate symptoms and improve quality of life. The lack of consistent endpoints for such trials has made cross study comparison difficult and has led to contradictory conclusions. The International Bone Metastases Consensus Working Party was established to create a standard set of endpoints and recommendations for future clinical trials. Recommendations were included regarding eligibility criteria, pain assessments, follow-up assessments, timing, as well as radiation techniques. Suggestions were also made to facilitate the ease with which different studies could be compared as well as to encourage widespread consistency in certain aspects of trial design. Investigators conducting clinical trials in bone metastases should continue to adopt these recommendations to ensure consistent guidelines based on the most recent literature.

## Introduction

### Bone metastases

Bone metastases are a frequent complication in patients with advanced cancer [1, 2]. Morbidity related to bone metastases is significant and includes pathologic fractures and spinal cord compression that subsequently reduce quality of life (QOL) [2-4]. Treatments for bone metastases are palliative, often with the intent of reducing the commonly associated pain [2, 4]. Radiotherapy in particular is commonly prescribed as an efficacious and efficient method to reduce symptoms associated with bone metastases.

## International Consensus on Palliative Radiotherapy Endpoints in Clinical Trials in Bone Metastases

Clinical trials have lent much to the understanding of external beam radiotherapy as an effective method for treating bone metastases [5]. Previous trials [5-7] evaluating the use of different dose fractionations have reported varying degrees of benefit gained from treatment which may be attributed to the lack of consistent study endpoints and endpoint definitions. For example, the RTOG evaluated data from their large randomized trial twice, once including only pain scores [8], and again, including both pain analgesic scores and the use of retreatment [9]. The authors concluded that both fractionated and single treatments were equally efficacious in the case of the former, but when analgesic scores and retreatment were included, the protracted fractionation schedules were of greater benefit in complete response analysis. In another randomized trial, Kirkbride et al. compared the efficacy of 8 Gy in 1 fraction vs. 20 Gy in 5 fractions for the palliation of bone metastases in a phase III randomized setting, use of analgesics was recorded along with pain scores (which had been used as a standard since) [10]. In the primary analysis, assessing pain scores alone, more significant pain relief was observed in the group who received hypofractionated radiotherapy regimens. However, when including analgesic intake, no significant difference in response was observed between the two regimens [10], which has been confirmed by numerous randomized trials and compiled in three meta-analyses [5-7].

As clinical trial design is subject to a variety of factors including study purpose, investigator preference, resources and personnel, two studies conducted for a similar purpose may diverge significantly at various stages. To maximize the opportunity for cross study comparison and consistency, the International Bone Metastases Consensus Working Party was established with the goal of producing unified endpoint definitions and recommendations for the design of future clinical trials in bone metastases.

### International consensus (2002)

The 2002 publication detailed the development of such endpoints and recommended specific standardized criteria, such as the inclusion of formal definitions of response rate that incorporated analgesic uptake (Table 1) [11]. Additionally, suggestions were made to aspects of clinical trial design for bone metastases such as eligibility criteria, pain assessments, follow-up assessments and timing as well as radiation techniques.

**Table 1:** Response Categories

Term	Definition
Complete Response	A pain score of zero at treated site with no concomitant increase in analgesic intake (stable or reducing analgesics in daily oral morphine equivalent (OMED))
Partial Response	Pain reduction of 2 or more at the treated site on a 0 - 10 scale without analgesic increase, or Analgesic reduction of 25% or more from baseline without an increase in pain.
Pain Progression	Increase in pain score of 2 or more above baseline at the treated site with stable OMED, or An increase of 25% or more in OMED compared with baseline with the pain score stable or 1 point above baseline
Indeterminate Response	Any response that is not captured by the complete response, partial response or pain progression definitions.

At the time of publication, directives for future research included: assessing the validity of proxy data, emphasis on developing accurate survival prediction methods, validation of bone metastases-specific quality of life (QOL) assessments, as well as investigating the cost-effectiveness of treatment [11]. Following the publication of the original consensus paper, several authors incorporated the updated endpoint definitions in their respective clinical trials [12-15]. Despite the fact that the incorporation of analgesic intake into response rate criteria resulted in a decrease in overall response rate, the data proving pain relief equivalency between single versus multiple fraction regimens has been consistently reported [1].

### Update of the international consensus (2011)

To ensure that the previously developed guidelines reflect the most recent evidence, the group published an update to the original 2002 consensus paper in 2011 [1]. Additional recommendations were made to facilitate cross study comparison and consistency by encouraging the use of well-established and well-defined endpoints, along with various suggestions and modifications to certain aspects of study design.

**Figure 1:**
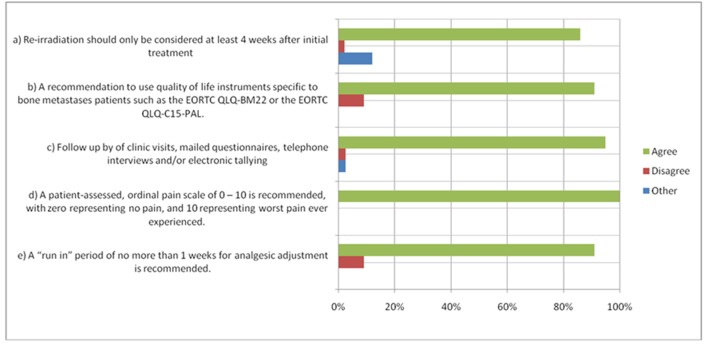
Items with > 80% Agreement. *Items with > 80% agreement as per the 2011 International Consensus. For other items, please see publication (1).

The updated consensus included previously unaddressed points such as the recommendation of specific QOL instruments, the allowance of proxy-rated data and a well-defined re-irradiation timeline, amongst other items. Some recommendations were deemed ‘minimum features’ of a bone metastases clinical trial and included the use of explicitly defined anchors on ordinal pain scale, offering electronic data capture methods for patients, utilization of proxy-rated data when necessary, and the use of validated QOL instruments specific to bone metastases. Items with >80% agreement are presented in Figure 1.

### Palliative radiotherapy side effects

As improvement of QOL is key for patients undergoing palliative radiotherapy for bone metastases, two key side effects have been discussed during the composition of the International Consensus - pain flare and radiation induced nausea and vomiting (RINV).

Palliative radiotherapy is typically associated with few treatment-related side-effects, most of which are conditional on the site that is irradiated [16]. A common side effect of patients undergoing palliative radiotherapy for bone metastases is pain flare defined as a temporary increase in bone pain immediately after radiotherapy [17, 18]. The transient increase in pain may subsequently reduce quality of life for the few days after treatment. Patients are usually directed to commence intake of ‘break-through’ pain medications on onset of pain flare, however, opioids do not prevent the phenomenon altogether and patients would still experience some degree of pain. The steroid dexamethasone is currently undergoing a randomized phase III study for the prophylaxis of pain flare in Canada. The phase II studies that have been completed since have shown promise, reducing the number of patients experiencing pain flare from approximately 2/5 to 1/5 [17, 18]. Patients undergoing palliative radiotherapy should be made aware of this phenomenon and be instructed regarding preventative or management strategies.

Despite being commonly associated as a side effect of chemotherapy, radiation induced nausea and vomiting can affect a significant percentage of patients depending on the areas irradiated [19]. Along with reductions in QOL, RINV can impair both functional and physical status [20] and should be proactively managed especially in high risk patients. Presutti et al. assessed the pattern of RINV in a palliative radiotherapy clinic, and found that approximately half of their sample experienced RINV, which occurred up to 10 days post-treatment [21]. Although the prevalence of this side effect is quite high, there exist few trials which have assessed management strategies for RINV. Salvo et al. conducted a systematic review of randomized trials examining the prophylaxis of RINV using 5-hydroxythryptamine-3 serotonin receptor antagonists (5-HT3 RAs) [19]. Although evidence exists supporting the use of 5-HT3 RAs for emesis, there is little data to support prophylaxis of nausea; even fewer data existed concerning QOL outcomes, adverse effects, or need for other rescue medicines. The authors concluded that further trials were necessary to investigate this issue in patients undergoing palliative radiotherapy.

## Future Directions

Steps towards the recommendations made will maximize the ability of future clinical trials to produce consistent data that can be easily interpreted and compared against other trials. However, early adoption of recommended guidelines can be limited by the lack of appropriate instruments. Guidelines, in turn, should be updated periodically to reflect both tools and technologies that are newly available.

### Bone specific quality of life instruments

As treatment aims shift towards palliation, QOL endpoints are important in quantifying the efficacy of an intervention. To effectively measure such an endpoint, change or improvement to QOL must be well defined. Furthermore, instruments must be able to capture meaningful QOL changes [22]. To be able to capture even the most subtle QOL changes relevant to a population, disease specific QOL instruments should be used whenever possible [23].

Prior to the development of a module specific to patients with bone metastases, quality of life issues were assessed most commonly through the EORTC QLQ-C30, a general questionnaire for all cancer patients [24]. The need for a more disease-specific assessment became apparent, as patients expressed a lack of consideration for relevant concerns such as pathological fractures, mobility issues or functional impairment [25]. The EORTC QLQ-BM22 was developed as a module to assess quality of life issues specific to patients with bone metastases. The instrument is administered with the QLQ-C30 and consists of 22 items in 4 scale responses (painful sites, painful characteristics, functional interference and psychosocial aspects) [26].

The development of the QLQ-BM22 took place over 4 phases as outlined by the EORTC Quality of Life Group [27]. In phase 1, ideas were generated regarding QOL issues relevant to patients with bone metastases. This included literature searches and interviews of both patients and health care practitioners involved in the care of such patients in three countries. Phase 2 commenced after the most important issues in phase 1 were designed to EORTC guidelines (i.e. 4-point response scales and questions regarding the last week). A total of 170 patients from nine countries assisted in the testing of the QLQ-BM22 under phase 3. Patients were debriefed and asked if any items were confusing, upsetting or intrusive, and to comment on whether or how a question should be altered if they found so. International field testing was conducted in 7 countries, and the results published in August 2011 confirmed the instrument’s reliability, validity and sensitivity in assessment of health related quality of life issues. Well-received by test centres and patients, the disease specific tool is now recommended for use in clinical trials assessing quality of life issues for patients with bone metastases [1].

The BOMET-QOL assessment is another health related QOL tool available to assess the experience of bone metastases patients [28]. Consisting of 10 questions scored on 5 point scales, the focus of this instrument differs from that of the BM22 in that it specifically addresses issues of pain and health related quality of life (HRQOL) deficits, while the BM22 encompasses additional factors such as treatment effects and psychosocial factors [29].With the advent of recently developed and validated tools that are disease specific to bone metastases, it is recommended that future trials employ such tools in conjunction with existing, general QOL measures [26].

### Follow-up of advanced cancer patients

Although self-rated patient data is ideal in clinical trials, it may become increasingly difficult for patients to complete assessments due to disease progression. This, in turn, can lead to poor data quality, reduce the ability to draw strong conclusions, or limit the ability to conduct analysis altogether [30]. To prevent significant drop-offs in data collection due to attrition, researchers have recommended the use of proxy data collection when the patient feels too tired or ill to do so. According to the 2011 consensus update, 72% of international experts would agree with collecting pain scores by proxy if the patient was too ill to complete the self-assessment [1].

While congruency between patient and proxy rated data varies depending on the population studied and the assessments used [31-33], proxy-rated data may offer valuable information in disease stages that would otherwise not be captured. Proxy validation for the EORTC QLQ-C30 has previously been demonstrated, showing good convergence between patient and proxy ratings at QOL scale extremities; for intermediate QOL ratings, however, more disagreement was observed [31]. As the disease specific module QLQ-BM22 has only recently been validated, next steps may include validation for proxy use to minimize the deleterious effects on data collection that come with patients’ declining cognitive and physical status.

Just as the development of more sensitive assessments aid the clinical trial process, new technologies should also be considered for use in clinical trial design. As technologies such as electronic data capture (EDC) and interactive voice recognition systems (IVRS) become more widespread and familiar to patients and physicians, clinical trial design should be adapted accordingly to improve the quality and efficiency of data collection. These methods, which shift away from the use of paper forms, have been shown to be cost-effective and patient friendly [34, 35]. Bliven et al. validated the use of computer software to administer similar health related QOL questionnaires and found high correlation between scores collected through electronic and paper methods [35]. Furthermore, they found that over 80% of patients preferred the online assessment to pen and paper methods; demographic factors such as computer literacy, age, and education level did not significantly impact completion of assessments. As such, EDC has been included as an option by which patients can choose to complete assessments [26].

Although the validity of data collected through EDC must be shown for the specific assessment and population in study, a number of studies involving cancer populations have shown good reliability and consistency between electronic and paper formats of testing [36-38]. IVRS represents another promising method of data capture, employing recorded, automated messages to deliver assessments [39]. Like electronic data capture, IVRS has shown good validity and reliability when compared to assessments completed in paper [40].

While the recommendations of employing electronic data capture are in response to patient preference, the utilization of proxy-rated data represents another way to alleviate patient burden by allowing a proxy to complete assessments on the patient’s behalf. To further reduce the burden placed on patients, condensed assessments created specifically for palliative populations are recommended, rather than their full length counterparts [1, 25]. For example, the EORTC QLQ-C15-PAL is a shortened core instrument used to study QOL concerns in palliative cancer patients, and has been described in multiple studies as more practical for palliative populations, resulting in better compliance rates [41]. Although the EORTC QLQ-C15-PAL represents a condensed version of the longer instrument, the information captured is both detailed and meaningful. Caissie et al. observed trends for certain cancer populations based on item responses; they found that patients who had bone metastases reported worse pain scores than others, while those with lung and brain cancers had more issues with fatigue [42].

## Conclusion

The International Consensus, updated in 2011, provides the means to facilitate cross study comparison and effective data collection amongst patients with bone metastases being treated with radiotherapy. A major advance provided by the International Consensus is the recommendation of specific endpoints, to ensure data from similar studies is accurate to compare. The defined endpoints created by the International Consensus have been adopted by the American Society for Radiation Oncology (ASTRO), who echo the importance of using these defined endpoints in future studies [43]. Future directions to facilitate cross study comparison include using bone metastases specific quality of life tools, such as EORTC QLQ-BM22 with QLQ-C30, or BOMET-QOL. To relieve patient burden and optimize patient participation, the use of a proxy, electronic data capture and interactive voice recognition systems, and using short, specific quality of life assessments are recommended.
